# Field-induced vortex-like textures as a probe of the critical line in reentrant spin glasses

**DOI:** 10.1038/s41598-021-99860-2

**Published:** 2021-10-21

**Authors:** N. Martin, L. J. Bannenberg, M. Deutsch, C. Pappas, G. Chaboussant, R. Cubitt, I. Mirebeau

**Affiliations:** 1 Université Paris-Saclay, CNRS, CEA, Laboratoire Léon Brillouin, 91191 Gif-sur-Yvette, France; 2 Faculty of Applied Science, Delft University of Technology, 2629 JB Delft, The Netherlands; 3 CNRS, CRM2, Université de Lorraine, Nancy, France; 4 Institut Laue Langevin, BP156, 38042 Grenoble, France

**Keywords:** Condensed-matter physics, Magnetic properties and materials

## Abstract

We study the evolution of the low-temperature field-induced magnetic defects observed under an applied magnetic field in a series of frustrated amorphous ferromagnets (Fe$_{1-x}$Mn$_{x}$)$_{75}$P$_{16}$B$_{3}$Al$_{3}$ (“a-Fe$_{1-x}$Mn$_{x}$”). Combining small-angle neutron scattering and Monte Carlo simulations, we show that the morphology of these defects resemble that of quasi-bidimensional spin vortices. They are observed in the so-called “reentrant” spin-glass (RSG) phase, up to the critical concentration $x_{\mathrm{C}} \approx 0.36$ which separates the RSG and “true” spin glass (SG) within the low temperature part of the magnetic phase diagram of a-Fe_1−x_Mn_x_. These textures systematically decrease in size with increasing magnetic field or decreasing the average exchange interaction, and they finally disappear in the SG sample ($x = 0.41$), being replaced by field-induced correlations over finite length scales. We argue that the study of these nanoscopic defects could be used to probe the critical line between the RSG and SG phases.

## Introduction

The role of disorder is central in condensed matter physics, as it favors the nucleation of defects which play a crucial role in the evolution and functionalities of a large variety of systems. Examples are magnetic vortices in type-II superconductors, skyrmions in helical magnets, Taylor cells in liquid flows, or twist grain boundary phases in cholesteric liquid crystals. Quite generally, defects allow new properties to penetrate in the system by forming intermediate states of matter, precursors of a phase transitions. In this context, we study here the influence of nm-size magnetic defects on the evolution from ferromagnetic (FM) and spin glass (SG) ground states. SG are archetypal disordered magnetic systems that have mobilized a large and continuous attention for decades. Their physics is mainly driven by atomic disorder *and* random sign interactions. In this work, we focus on the so-called “reentrant” spin glasses (RSG), where a mixture of FM and antiferromagnetic (AFM) couplings (usually tuned by the concentration of magnetic ions and, in some cases, heat treatment) leads to SG-like irreversibilities at low temperatures, deep inside the FM ordering region. In RSGs, the nature of the coexistence between SG behavior and ferromagnetism has been much debated. Here, we show that the observation of magnetic field-induced vortex-like textures, although not predicted by current theories, could be a key point to distinguish between RSG and SG ground states at a microscopic level.

Historically, the RSG and SG ground states have been described by two concurrent theoretical approaches. The infinite range mean field (MF) picture yields a phase diagram with a tricritical point and a vertical line between RSG and SG phases^1^. Below this line, *i.e.* in the weakly frustrated case, lowering temperature leads to the occurrence of mixed RSG phases, where SG and FM order parameters coexist microscopically. At each magnetic site, “longitudinal” spin component, forming a long-range magnetic order (LRMO), coexist with a “transverse” one, randomly oriented in the perpendicular plane. Alternatively, random field (RF) arguments predict the breakdown of LRMO for an arbitrarily small amount of disorder in dimensions $d \le 4$, as formalized by the Imry-Ma (IM) argument^2^. This argument was used together with percolation approaches to describe the RSG phase as randomly oriented clusters spatially separated from the “infinite” FM one. The latter would break due to RFs at the RSG-SG threshold, in a cross-over transition^3,4^. The IM argument was recently complemented by a series of Monte-Carlo (MC) simulations suggesting that, in the case of ferromagnets exposed to a RF, the IM domains are protected against a full collapse of the magnetization by the nucleation of topological defects, such as pinned hedgedhogs in 3 dimensions^5^ or a “skyrmion-antiskyrmion glass” in 2 dimensions^6^.

On the experimental side, magnetic defects -akin to nm-size vortices- have been observed by small-angle neutron scattering (SANS) in weakly frustrated RSGs under an applied magnetic field. The family of studied compounds (Ni$_{1-x}$Mn$_{x}$, Au$_{x}$Fe$_{1-x}$, Fe$_{1-x}$Al$_{x}$, or a-Fe$_{1-x}$Mn$_{x}$) includes different types of disorder, magnetic interactions and sample form (single crystal, polycrystal or amorphous samples)^7–10^. In all cases, SANS experiments show that the transverse spin components rotate over a finite length scale which defines the average size of the vortex-like spin textures. These data, supported by MC simulations^11^, also indicate that they shrink with increasing the applied field, but their behaviour at strong frustration and across the RSG-SG threshold has not been studied so far.

In order to address this point, we focus here on the series of frustrated amorphous ferromagnets (Fe$_{1-x}$Mn$_{x}$)$_{75}$P$_{16}$B$_6$Al$_3$ (“a-Fe$_{1-x}$Mn$_{x}$”). a-Fe$_{1-x}$Mn$_{x}$ maps a case of 3d disordered Heisenberg spins, where frustration can be chemically tuned through the competition of FM and AFM interactions. Using SANS, we follow the evolution of the field-induced vortex-like spin textures with increasing frustration, as the magnetic ground state evolves from RSG to SG. We show that these non-singular defects are characteristic of the RSG ground state. Their average size obeys scaling laws up to the critical concentration and maximum applied field. They eventually disappear above the RSG-SG threshold, showing that a non-zero average exchange is needed for their stabilization. Our results may open a route to reconcile the MF and RF pictures of the RSG state, opposed for decades. Indeed, such nm-size magnetic defects can probe the nature of the frustrated medium and be the fingerprints of a quantum phase transition at a microscopic level. Our experimental study is also supported by Monte Carlo simulations, which show that the occurrence of these defects, as well as their evolution as a function of magnetic field, can be globally reproduced using a very limited amount of ingredients. These results suggest that frustration and disorder can be used to engineer the average size of individual defects in a controlled and reproducible way in disordered frustrated ferromagnets.

## Samples and their macroscopic magnetic properties

The a-Fe$_{1-x}$Mn$_{x}$ system is perfectly suitable for our study. Its macroscopic properties and transition temperatures are well known and almost independent of the sample synthesis, while the amorphous character guarantees the absence of structural defects which could otherwise act as pinning centers^12^. Frustration is monitored by the Mn concentration *x* which controls the relative amounts of AFM Mn-Mn nearest neighbor (NN) bonds with respect to the FM Mn–Fe and Fe–Fe ones. Here, we study seven RSG samples of concentrations ranging from $x = 0.22$ to $x = 0.35$ and a SG sample with $x = 0.41$, previously studied by Mössbauer spectroscopy, neutron depolarization and muon spin rotation^13,14^ ($\upmu$SR). Samples were prepared using the “wheelbarrow” technique and their amorphous nature was checked using neutron diffraction (see Supplementary Information with Supplementary Figures S1 and S2). The resulting ribbons, of typical thickness $\approx$ 30–70 $\upmu$m and $\approx$ 8–10 mm width, can be easily cut or piled-up to perform magnetic and neutron scattering experiments.

**Figure 1:**
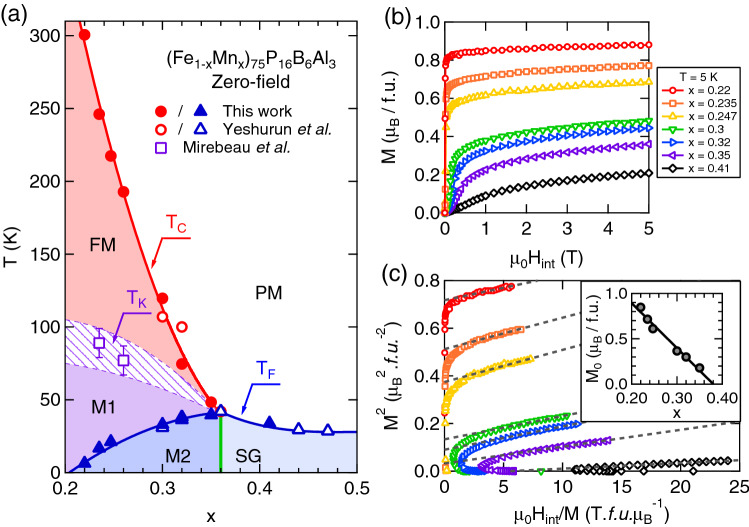
Phase diagram and magnetization of a-Fe$_{1-x}$Mn$_{x}$—(**a**) Zero-field magnetic phase diagram of a-Fe$_{1-x}$Mn$_{x}$ inferred from magnetic ac-susceptibility. For $0.2 \lesssim x \lesssim x_\text{C}$, cooling from the high-temperature FM state leads to a sequence of two mixed states: M1, involving the freezing of transverse spin components^18^ and M2, where replica symmetry is spontaneously broken (i.e., an analog of the SG state). Above $x = x_\text{C} \approx 0.36$, the ferromagnetic (FM) phase is suppressed and replaced with a “canonical” spin-glass (SG) state at low temperature. Open symbols are data from Mirebeau et al.^13,14^ and Yeshurun et al.^15^ which are added to our results for $T_\text{C}$ and *T*$_\text{F}$ for the sake of completeness. The green vertical line indicates a putatively vertical critical line between the RSG (M2 phase) and SG regimes^1^. (**b**) Low-temperature magnetization curves of a-Fe$_{1-x}$Mn$_{x}$. (**c**) Arrot plots computed from the data of panel (**b**). Dashed lines are linear fits to the high-field data. Inset shows the *x*-dependence of the spontaneous magnetization $M_\text{0} = M(\upmu _{0}H_\text{int} \rightarrow 0)$.

The zero-field magnetic phase diagram of a-Fe$_{1-x}$Mn$_{x}$ is shown in Fig. 1a. In the RSG regime, i.e. for small *x*, a transition from the high temperature paramagnet to the low temperature FM state occurs at the Curie temperature $T_\text{C}$. The latter decreases upon increasing *x*, up to a tricritical point located at $x = x_\text{C} \approx 0.36$ (as determined by previous magnetic susceptibility^15^ and neutron depolarization^14^ measurements). At this point, the $T_\text{C}$ line meets another transition line, corresponding to a spin glass freezing of the system upon cooling below $T_\text{F}$. The low-temperature part of the phase diagram is then ruled out by *strong irreversibilities* of the magnetization, in both the M2 phase (for $x < x_\text{C}$) and the “pure” SG state (for $x > x_\text{C}$). Although they present similar low-field macroscopic magnetic properties, it is important to note that the M2 and SG phases are fundamentally different. Indeed, M2 is a “mixed” phase, within which FM and SG orderings coexist at a microscopic scale, while the FM order is lost in the “pure” SG state. We inferred the $T_\text{C}$ and $T_\text{F}$ of our samples from sharp increases and decreases of the real part of the ac-susceptibility (see Supplementary Information with Supplementary Table S1 and Supplementary Figures S4, S5 and S6) and compared them with the results of Yeshurun et al.^15^ in Fig. 1a.

For $x \le x_\text{C}$, an additional transition takes place at the so-called “canting” temperature $T_\text{K}$, situated between $T_\text{C}$ and $T_\text{F}$ as predicted by the MF model of Gabay and Toulouse^1^. It corresponds to the freezing of the *transverse* spin components and the onset of *weak irreversibilities* throughout the intermediate mixed M1 phase^16^. $T_\text{K}$ is however not visible in our zero-field ac-susceptibility data, since its observation requires distinguishing between transverse and longitudinal components of the magnetic moments. Signatures of $T_\text{K}$ have been reported in a-Fe$_{1-x}$Mn$_{x}$ by comparing magnetization with either Mössbauer spectroscopy^13^ or $\upmu$SR^14^ data (see Fig. 1a). This canting temperature has been identified as the temperature border, above which the vortex-like textures described in the present work vanish^7,10^ and below which dynamical anomalies, such as damping of the FM spin waves, occur^17^.

We also performed dc-magnetization measurements in the 0-5 T field range in order to verify that all samples with $x < x_\text{C}$ retain a ferromagnetic character, while the field at which technical saturation takes place increases with *x* under the effect of increasing magnetic frustration (Fig. 1b). From the corresponding Arrott plots (Fig. 1c), we also deduced the *x*-dependence of the spontaneous moment $M_\text{0} = M(\upmu _{0}H_\text{int} \rightarrow 0)$. As shown in the inset of Fig. 1c, this leads to an extrapolated zero moment for $x = 0.38(3)$, which is consistent with the literature value of the critical concentration $x_\text{C}$ where long-range FM order is lost.

## Small-angle neutron scattering (SANS)

We have carried out a small-angle neutron scattering (SANS) experiment at the PAXY beamline (Orphée Reactor, Saclay, France). A horizontal magnetic field $\upmu _{0}{\mathbf {H}}$ up to 4 T was applied transverse to the beam direction (i.e., in the detector plane). All data were obtained in the zero field cooled (ZFC) state at *T* = 3 K. This temperature was chosen because (i) it is well-below $T_\text{C}$ and $T_\text{F}$ for all samples (Fig. 1), and (ii) allows neglecting the contribution of phonons and magnetic excitations (spin waves) to the SANS patterns. Data were corrected and calibrated as described in the Supplementary Information with Supplementary Tables S2 and S3.

**Figure 2:**
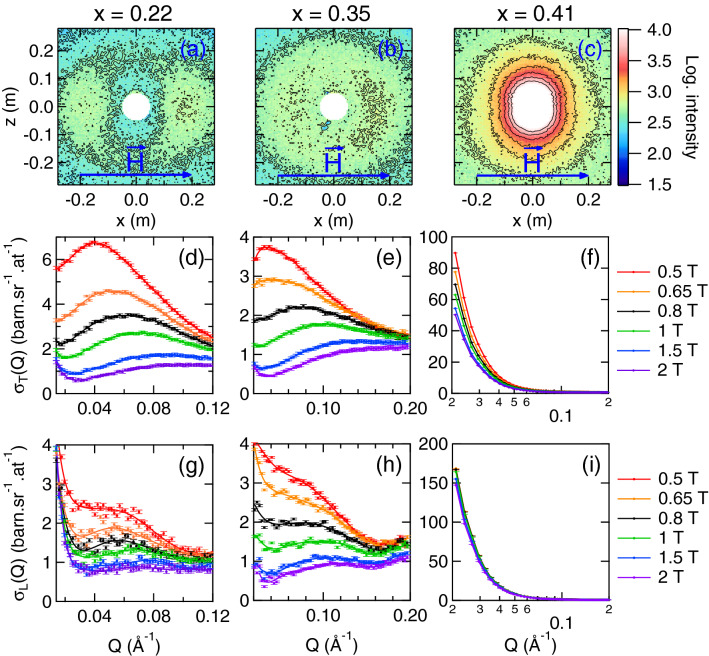
SANS data on a-Fe$_{1-x}$Mn$_{x}$ in the M2 and SG phases—(**a–c**) Scattering maps recorded at $T = 5$K under an applied magnetic field of 1.5 T. (**d–f**) Field-dependence of the transverse magnetic cross section $\sigma _\text{T}(Q)$ (**g-i**) Field-dependence of the longitudinal magnetic cross section $\sigma _\text{L}(Q)$. The values of the applied magnetic field are indicated for panels (**d–i**).

Typical SANS patterns are shown in Fig. 2, for compositions *x* respectively below (Fig. 2a,b) and above (Fig. 2c) $x_\text{C}$. One can immediately note differences between these two cases. On the one hand, maxima of intensity appear parallel to the applied field at a finite value of the momentum transfer *Q* when $x < x_\text{C}$. On the other hand, SANS from the $x = 0.41$ sample is typical of field-induced ferromagnetic-like correlations centered at $Q = 0$. In order to separate contributions of magnetic moments transverse (*T*) and longitudinal (*L*) to the applied field, we make use of the neutron selection rule which states that only components perpendicular to the scattering vector ${\mathbf {Q}}$ contribute to the observed scattering cross section $\sigma ({\mathbf {Q}})$. This translates into the following relations

1 $$\begin{aligned} \sigma _\text{T}\left( {\mathbf {Q}}\right) + \sigma _\text{bg}\left( {\mathbf {Q}}\right) /2 = {\tilde{\sigma }}\left( {\mathbf {Q}}\parallel {\mathbf {H}}\right) /2 \quad \text {and} \quad \sigma _\text{L}\left( {\mathbf {Q}}\right) + \sigma _\text{bg}\left( {\mathbf {Q}}\right) /2 = {\tilde{\sigma }}\left( {\mathbf {Q}}\perp {\mathbf {H}}\right) -{\tilde{\sigma }}\left( {\mathbf {Q}}\parallel {\mathbf {H}}\right) /2 , \end{aligned}$$

where $\sigma _\text{bg}$ denote the background contributions from the sample (*e.g.*, nuclear scattering) and ${\tilde{\sigma }}$ the full observed scattering within sectors of 60$^{\circ }$ opening angle, parallel or perpendicular to ${\mathbf {H}}$. Therefore, a radial integration of the SANS data along the horizontal and vertical direction allows retrieving the *Q*-dependences of $\sigma _\text{T}$ and $\sigma _\text{L}$ independently, assuming an isotropic $\sigma _\text{bg}$.

The result of such procedure is shown in Fig. 2d–i. In the weakly frustrated $x = 0.22$ RSG sample, the intensity is clearly enhanced along the field direction, i.e. for ${\mathbf {Q}}\parallel {\mathbf {H}}$, showing that the contribution of spin components transverse to the magnetic field are dominant in the explored *Q*-range, whereas the opposite behavior is observed in the $x = 0.41$ SG sample. As a general feature, we observe field-induced peaks in $\sigma _\text{T}(Q)$ at $Q = Q_\text{max}$ for all compositions $x < x_\text{C}$. $Q_\text{max}$ moves to higher values when the field increases at constant *x*, and also shows a systematic stiffening as *x* increases towards $x_\text{C}$. $\sigma _\text{L}(Q)$ shows a broad maximum, but it is more difficult to point because its intensity is much smaller.

In what follows, we focus on the transverse cross section $\sigma _\text{T}(Q)$, which is defined in the most general case as

2 $$\begin{aligned} \sigma _\text{T}\left( {\mathbf {Q}}\right) \propto \langle F_\text{T}^{2}(Q) \rangle - \langle F_\text{T}(Q) \rangle ^{2} \left[ 1-S_\text{int}(Q)\right] , \end{aligned}$$

where $F_\text{T}(Q)$ is the form factor of the transverse defects and $S_\text{int}(Q)$ is an interference function that expresses the local correlations between two such defects. Assuming that the latter are organized in a liquid-like order ($S_\text{int}(Q) \rightarrow 1$) and noting that the form factor of a “regular” bidimensional vortex is null ($\langle F_\text{T}(Q) \rangle = 0$)^19,20^, $\sigma _\text{T}(Q)$ is simply proportional to $\langle F_\text{T}^{2}(Q)\rangle$.

**Figure 3:**
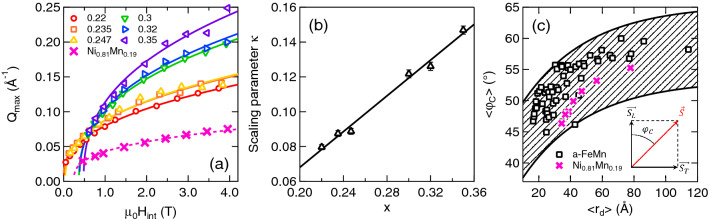
Scaling of the properties of the vortex-like textures—(**a**) Field-dependence of the position $Q_\text{max}$ of the maximum in $\sigma _\text{T}(Q)$ for each studied compositions. Solid lines are results of a global fit of Eq. 3 to the data. (**b**) *x*-dependence of the scaling parameter $\kappa$, extracted from a fit of Eq. 3 to the data of panel (**a**). (**c**) Average canting angle $\langle \varphi _\text{C} \rangle$ as a function of the average radius of the vortex-like textures $\langle r_\text{d} \rangle$. In panel (**a**, **c**), data for Ni$_{0.81}$Mn$_{0.19}$ is shown for comparison (pink crosses)^20^. Dashed pink line is the result of a fit of Eq. 3 to the data.

In the whole $x < x_\text{C}$-range, the field-dependence of $Q_\text{max}$ obeys a scaling law of the form

3 $$\begin{aligned} Q_\text{max} (\upmu _\text{0}\,H_\text{int},x) = \kappa (x) \, \left[ \upmu _\text{0}\,\left( H_\text{int} - H_\text{0}(x)\right) \right] ^\gamma , \end{aligned}$$

where $H_\text{0}(x)$ is a composition-dependent saturation field, increasing with *x*. A global fit of Eq. 3 to the data yields a unique exponent $\gamma = 0.39(1)$ and *x*-dependent scaling parameters $\kappa (x)$ (Fig. 3a,b). Results previously obtained on Ni$_{0.81}$Mn$_{0.19}$ are also reported in Fig. 3a. In this case, a fit of Eq. 3 to the data yields an exponent $\gamma = 0.34(2)$, quite close to the value derived for the a-Fe$_{1-x}$Mn$_{x}$ series.

In order to account for this scaling law, we propose a simple picture and provide a physical meaning for its parameters. First, we note band structure calculations of dilute FeMn alloys^21–23^ where Mn-Mn NN interactions are AFM whereas Fe-Fe and and Fe-Mn NN interactions are FM. Based on previous SANS results on a crystalline Ni$_{0.81}$Mn$_{0.19}$ sample, as well as toy models and MC simulations^20^, we interpret $\sigma _{T}(Q)$ by assuming uncorrelated defects, akin to nm-size vortex-like textures, nucleated around Mn-Mn first neighbor pairs. In the simplest picture, the spin components $M_\text{T}$ are ferromagnetically correlated and rotate over an average radius $\langle r_d \rangle = \pi /Q_\text{max}$ to compensate the transverse magnetization inside the vortex-like textures^20^.

Using this picture, we can readily interpret the evolution of the SANS patterns with magnetic field and Mn concentration *x*. At a given *x*, $\langle r_d \rangle$ decreases with increasing the magnetic field (Fig. 3a), as the spins composing the defects progressively align along the field, albeit not necessarily in a uniform fashion. Their gradual suppression yields a slow increase of the magnetization, taking place in the magnetization quasi-plateau (Fig. 1b). At a given field, $\langle r_d \rangle$ also decreases with an increase of *x*, which governs the concentration of AFM NN pairs within the samples. As we will show below, these features translate the weakening of the average exchange interaction $\langle J \rangle$.

Altogether, such scaling law suggests that the characteristic size of the defect is governed by the ratio between the magnetic field and the average exchange interaction. To check this picture in more details, we have searched for a common law governing the bulk magnetization curves *M*(*H*) at 5 K in the a-Fe$_{1-x}$Mn$_{x}$ system. From the experimental curves, we find a $M \approx \left( \upmu _{0}\,\left[ H_\text{int}-H_\text{0}\right] \right) ^{1/3}$-dependence above a threshold field value $H_\text{0}$, which scales with the saturation field deduced from the magnetization curves (see Supplementary Information with Supplementary Figure S3). We can compare this dependence with that observed in ferromagnets close to saturation, where the magnetic field suppresses micro-structural defects. Here the magnetostatic exchange length $\Lambda$ which controls the defect size is defined as^24^

4 $$\begin{aligned} \Lambda = \left( \frac{2A}{\upmu _0 M^2}\right) ^{1/2}, \end{aligned}$$

where *A* is the exchange stiffness and *M* the bulk magnetization. Identifying *A* with the average exchange term $\langle J \rangle$ and $\Lambda$ with $\langle r_\text{d} \rangle$ leads to the following dependence for $Q_\text{max}$

5 $$\begin{aligned} Q_\text{max} \approx \frac{\left( \upmu _{0}\,\left[ H_\text{int}-H_\text{0}\right] \right) ^{1/3}}{\langle J \rangle ^{1/2}}. \end{aligned}$$

which is quite close to the dependence found experimentally (Eq. 3), noticing that the experimental value of the exponent $\gamma =0.39 (1)$ is slightly above the value 1/3 from macroscopic magnetization. This comparison however confirms that the average exchange interaction and the applied magnetic field are the main ingredients needed to control the behaviour of the observed magnetic defects, although additional anisotropic exchange terms (such as the Dzyaloshinskii-Moriya interaction) could play a minor role. A perfect mapping of the two cases is in fact not expected, especially for the strongly frustrated RSGs, where the magnetization does not show any clear saturation plateau. Taking this analysis into account, one can however tentatively evaluate the average exchange constant from the scaling parameter $\kappa$ by $\langle J \rangle =\kappa ^{-1/\gamma _\text{eff}}$, with $1/3 \le \gamma _\text{eff} \le 0.39$.

We can further define the canting angle $\langle \varphi _\text{C} \rangle$, averaged over the size of the vortex-like textures, by the expression

6 $$\begin{aligned} \langle \varphi _\text{C} \rangle = \frac{\text {arctan}\left( \langle M_\text{T}^2 \rangle / \langle M_\text{L}^2 \rangle \right) }{\langle r_\text{d} \rangle } \approx \text {arctan}\left( \frac{\sigma _\text{T}(q_\text{max})}{\sigma _\text{L}(q_\text{max})}\right) \cdot q_\text{max} . \end{aligned}$$

The result is shown in Fig. 3c, which illustrates the correlation between the average defect radius $\langle r_\text{d} \rangle$ and $\langle \varphi _\text{C} \rangle$. $\langle \varphi _\text{C} \rangle$ is maximum at low fields when $\langle r_\text{d} \rangle$ is the largest (around 120 Å), and reaches values of 55-60 degs. As for comparison, the canting angle deduced from the Mössbauer measurements of the $^{57}$Fe hyperfine field in the *x* = 0.235 sample, is around 35(7) degs, with a large distribution^13^. As the field increases, $\langle r_\text{d} \rangle$ decreases and the canting angles decreases as well, reaching values around 45 degrees at the smallest $\langle r_\text{d} \rangle$ $\approx$ 20 Å. To summarize this point, the average canting angle $\langle \varphi _\text{C} \rangle$ increases as the transverse spin components and magnetic disorder in the transverse plane increase. Corresponding data, extracted from our previous SANS experiment on Ni$_{0.81}$Mn$_{0.19}$ fall within the same range and follows a very similar trend^20^. This suggests that the observed vortex-like textures are relatively independent on the sample form (single crystalline or amorphous) and could represent an immanent feature of the large family of RSGs.

## Monte Carlo simulations

In order to get a deeper insight onto the properties of the magnetic defects evidenced in our SANS experiments, we have carried out a series of Monte Carlo simulations on 2d square lattices containing 10$^4$ spins. The model is described by the following classical Hamiltonian:

7 $$\begin{aligned} {\mathcal {H}} = - \sum _{ij} J_{ij} \, {\mathbf {S}}_{i} \cdot {\mathbf {S}}_{j} - \alpha H \sum _{i} S_{i}^{z} , \end{aligned}$$

where ${\mathbf {S}}_{i,j}$ are Heisenberg spins with $|{\mathbf {S}}_{i,j}| = 1$, $J_{ij}$ are NN exchange constants with $|J_{ij}| = 1$, $\alpha = \upmu _\text{B} / k_\text{B} \approx 0.672$ is a coupling constant and the magnetic field *H* is applied along the *z* direction. The first sum in Eq. 7 runs over NN pairs. All simulations started by generating spin configurations at a temperature $T/J = 2$, where a concentration *x* of “impurities” (i.e., analogs of Mn ions) are scattered *randomly* within an otherwise ferromagnetic matrix (i.e., analogs of Fe ions). The following rule is then applied to calculate the sign of the nearest-neighbors exchange terms: two nearest-neighbor impurities will be coupled antiferromagnetically ($J_{ij} = -1$) while all other pairs will be coupled ferromagnetically ($J_{ij} = 1$). This situation is meant to map quite closely the one expected from band structure calculations^21–23^, let alone the actual atomic connectivities. The key quantity describing the MC sample is therefore the concentration $c_\text{AFM}$ of AFM bonds (with $c_\text{AFM} = x^2$). In order to stick even more to the experimental situation, the system is slowly cooled down to $T/J = 0.01$ at $H = 0$ (in steps $H/J = 0.1$) and the field is further raised in steps $\Delta H/J = 0.01$. We shall show in the following that such a simple scheme allows for a “zero-order” simulation of the properties of the RSGs, and a reasonable description of the experimental observations reported in this paper.

**Figure 4:**
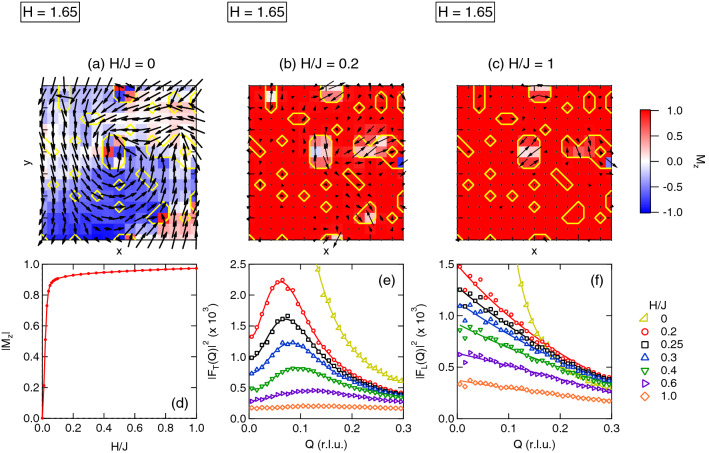
Spin configurations and Fourier analysis of the MC simulations—(**a–c**) Snapshot of a spin configuration ($15 \times 15$ spins) obtained on a $x = 0.23$ sample ($c_\text{AFM} \approx 0.05$) at $T/J = 0.01$ for (**a**) $H/J = 0$, (**b**) $H/J = 0.2$ (slightly above technical saturation) and (**c**) $H/J = 1$. Arrows represent the in-plane magnetization while color map shows the out-of-plane (*i.e.*, parallel to the applied magnetic field) component $M_\text{Z}$. Yellow lines border the regions where impurity spins are located, anchoring the field-induced localized vortex-like textures (see text). (**d**) Field-dependence of the average longitudinal magnetization $|M_\text{Z}|$. (**e, f**) Squared Fourier transforms of the transverse (**e**) and longitudinal (**f**) spin correlations for different values of *H*/*J*, here averaged over 40 realizations (see Methods and Supplementary Information with Supplementary Figures S9 and S10).

We now focus on the case of a weakly frustrated RSG sample ($x = 0.23$, leading to $c_\text{AFM} \approx 0.05$) to investigate the spin configurations and their corresponding Fourier transforms as the magnetic field increases (additional cases, displaying essentially similar behaviors, are addressed in the Supplementary Information with Supplementary Figures S9 and S10). In the zero-field or low field region, vortex-like structures are observed around AFM NN pairs, coexisting with domains walls of large length scale which separate the magnetic domains. As shown in Fig. 4a, these domain walls can involve transverse chiral components as well as local defects. However, a Fourier transform of the spin maps show that they *do not* yield a maximum of the scattered intensity as for the vortex-like structures, but rather a huge increase of the intensity at low *Q* values. As the field increases, these walls are rapidly suppressed, leading to a strong increase of the magnetization, and to the observation of isolated vortex-like defects. Such textures are nucleated randomly in the sample around AFM NN pairs (Fig. 4a–c), so that in zero (or small) applied field, they could form both in the ferromagnetic domains and in the domain walls (Fig. 4a). However, they are observed in the spin maps only when the field is high enough to suppress the contribution of the domain walls. In this regime, vortex-like textures emerge from the ferromagnetic *vacuum* while magnetization shows a quasi-plateau (Fig. 4d). This leads to a clear maximum in the squared Fourier transforms of the transverse magnetization $|F_\text{T}(Q)|^2$ at a finite *Q*value for intermediate and large applied magnetic fields (Fig. 4e). The defects shrink as the field increases further (and so, the peak in $|F_\text{T}(Q)|^2$ shifts towards larger Q values), and they slowly disappear together with the AFM pairs which nucleate them. The complete destruction of all AFM pairs should only occur at very high fields, much larger than the exchange interaction ($H \gg J$ ). To summarize, Fourier transforms of the spin maps in the region of the magnetization plateau shows features very similar to the experimental ones, both for the transverse and longitudinal contributions to the cross section (compare Figs. 2d,e,g,h and 4e,f). Finally, we note that while being resilient to very large applied fields, the vortex-like textures obtained in the simulations have a vanishing topological charge, most likely due to the ill-defined FM vacuum endowing them with irregular shapes. They are however characterized by a finite *vorticity*. This confirms the picture obtained using SANS, namely that of finite-size objects within which the transverse magnetization is compensated.

## Discussion

As our key experimental result, we have shown that in a frustrated ferromagnetic system, vortex-like defects are a characteristic feature of the RSG which are observable when the system approaches magnetic saturation. However, unlike flux lines in type-II superconductors or skyrmions in helical magnets, these objects do not form a lattice made of infinite length “tubes”. Instead, they resemble “pancakes” made of locally twisted spin configurations and are independent one another, as consistently shown by our SANS data and MC simulations. Their quasi-2d character is also supported by previous simulations performed on 3*d* spin maps^20^.

Their average size $\langle r_\text{d} \rangle$ decreases with decreasing the average exchange interaction, and the vortex-like textures disappear in the true SG, while $\langle r_\text{d} \rangle$ also decreases with increasing the applied magnetic field. The whole behaviour is captured by a scaling law governing $\langle r_\text{d} \rangle$, where the only ingredient is the ratio of the internal magnetic field to the average exchange interaction. Similar laws derived from magnetostatics govern the field behaviour of different macroscopic quantities. For example, one can quote the quasi saturated magnetization of ferromagnets with microstructral defects, the magnetization of type II superconductors, or the thickness of Bloch walls in ferromagnets.

In the RSGs, the presence of vortex-like defects up to the critical concentration, and their collapse in the SG phase when the average exchange interaction becomes smaller than the width of its distribution, strongly supports the existence of a critical line between RSG and SG regions. Our observations therefore support a MF description of the RSG phase diagram, rather than the crossover evolution towards FM breakdown predicted by random field arguments. We however recall that the original MF model of Gabay–Toulouse^1^, although being able to correctly describe the experimental (*x*, *T*)-phase diagram, cannot predict any defect, since the transverse spin component is randomly distributed in the transverse plane. The present observation can therefore help refining the current models for the RSG problem, by considering the observed magnetic microstructure.

Altogether, our SANS results combined with MC simulations suggest two complementary phenomena: (1) the vortex-like textures observed in both cases emerge from an average ferromagnetic medium acting as a vacuum field, required for their stabilization (and, hence, their experimental observation using SANS) and (2) they protect the ferromagnetic domains from breaking down under the influence of magnetic fustration. The MC calculations strongly suggest that the defects are nucleated around AFM NN pairs. In “real” samples, these pairs likely arise from Mn–Mn first neighbours, as suggested by band structure calculations in Fe–Mn^21–23^.

In the weakly frustrated RSGs, isolated nm-size vortex-like textures can be clearly distinguished from domain walls by combining magnetization, SANS and MC simulations. The domain walls recall those observed in non-frustrated ferromagnets, but they involve AFM bonds, which induce magnetic defects where the spin canting is locally enhanced, and which act as pinning centers at low temperature. Below $T_\text{F}$, this process leads to a strong decrease of the susceptibility $\chi (T)$ and to strong irreversibilities of the magnetization *M*(*H*). This picture is supported by electron microscopy^25^, neutron depolarisation^18^ and recent acoustic absorption measurements^26^, which clearly show that LRMO and $\upmu$m-sized domains are preserved in the ground state of the weakly frustrated RSGs. As the field increases, the domain walls are rapidly washed out, whereas isolated vortex-like textures persist up to much higher fields, where their contribution to the SANS can be clearly identified. In the highly frustrated RSGs, the distinction between these objects and the domain walls smears, since the average domain size decreases (becoming comparable to the domain wall thickness) and the magnetization plateau disappears. The vortex contribution is still clearly observed in the SANS data, in sharp contrast with the SG sample.

MC simulations are in turn crucial to refine the above picture, and already extend our results to a field range (or more , a *H*/*J* range) inaccessible to experiment. The good agreement between the Fourier transform of the MC spin maps and our experimental results should be noticed, considering that the simulated case is over-simplified with respect to the experimental one. We outline here that the MC spin maps show a huge amount of disorder around the local defects which nucleate the vortices. Considering the chemical disorder, many different types of vortices could a priori exist in the sample and they are indeed observed in the MC spin maps. However, their average size can be determined without ambiguity, as it leads to a maximum in *Q*-space, the position $Q_\text{max}$ of which is tuned by the *H*/*J* ratio.

To conclude, we briefly compare the above vortex-like textures with the topological defects observed in ferromagnets submitted to weak random fields. Quite generally, topological defects are expected when the number of spin components *n* is such that $n \le d+1$ where *d* is the dimension of space^27^, namely in all experimental cases. For instance, non-singular skyrmions with a finite topological charge are observed in the ($n = 3$, $d = 2$) case^5^. Their existence is a consequence of crossing points between lines where all RF field components cancel at the same time. This leads to the very interesting concept of “skyrmion glass”, composed of regions with oscillating positive and negative topological charges, and sizes scaling that of the IM domains^6^. Importantly, these defects, which prevent the magnetization from collapsing, should lead to a measurable topological Hall effect (THE). Conversely, the vortex-like defects stabilized by magnetic frustration (induced by competing interactions and bare interaction randomness) have a very small topological charge due to their very irregular shape, but they could also yield a peculiar Hall signal. In this context, it is worth noting that an anomalous Hall effect was actually predicted^28^ and observed in AuFe RSG or SG alloys^29,30^, as a probe of non coplanar (chiral) spin configurations. A quantitative study of the field-dependent Hall response of a-Fe$_{1-x}$Mn$_{x}$ above and below $x_\text{C}$ could refine the description of the RSG ground state, given that the defects involved in the two regimes likely have different natures.

## Materials and methods

### Materials

The amorphous samples of (Fe$_{1-x}$Mn$_{x}$)$_{75}$P$_{16}$B$_{3}$Al$_{3}$ (0.22 $\le$ x $\le$ 0.41) used in this study were prepared using the “wheelbarrow” technique, which consists in casting molten alloy with the desired composition on a spinning wheel. Being a strong neutron absorber, $^{10}$B was replaced with isotopic $^{11}$B. Samples were cut in foils of about 1 cm$^2$ surface with thicknesses varying from 30 to 70 $\upmu$m. These foils were piled up in order to increase the total sample thickness and yield a large enough sample mass for the small-angle neutron scattering experiments. Conversely, individual foils were cut into rectangular pieces, having a height to width ratio close to 2, for the magnetic measurements.

### Magnetic measurements

The ac-susceptibility of the a-Fe$_{1-x}$Mn$_{x}$ samples have been obtained using a Quantum Design Physical Properties Measurement System (PPMS, Dynacool 9 T, Laboratoire Léon Brillouin, France). Magnetization curves were measured using a Quantum Design Superconducting Quantum Interference Device magnetometer (SQUID, MPMS-XL 5 T, Technische Universiteit Delft, The Netherlands).

### Small-angle neutron scattering (SANS)

SANS experiments were performed on the PAXY instrument at the Orphée reactor (Laboratoire Léon Brillouin, Gif-sur-Yvette, France), operated in a standard pinhole geometry. Neutron wavelength was set to 4 and 6 Å, while keeping the sample-to-detector distance to 2.8 m. An horizontal magnetic field was applied using a cryomagnet (Oxford SM4000), allowing to reach fields of 10 T while cooling the sample down to 2 K. Additional SANS measurements were performed on the D33 instrument (Institut Laue Langevin, Grenoble, France)^31^, as described in the Supplementary Information with Supplementary Figures S7 and S8.

### Monte Carlo simulations

Monte Carlo simulations were carried out using the “adaptative” algorithm described by Alzate-Cardona et al. ^32^. 40 maps, containing 10$^{4}$ spins sitting on the vertices of a square lattice were generated at high temperature. In this model, each spin interacts with its nearest neighbors only (see Eq. 7). A concentration *x* of “impurity” spins was randomly scattered across the matrix in order to introduce AFM couplings ($J = -1$) within the FM matrix ($J = 1$). Each sample was cooled down to $T/J = 0.01$ in zero-applied field (in steps of $T/J = 0.1$), and the field was then increased in small steps ($H/J = 0.01$) to study the evolution of the spin configurations and their Fourier transforms. In order to properly equilibrate the system, 500 Monte Carlo steps were performed at each stage of the simulation (both during the zero-field cooling and the low-temperature field ramping). Note that Fig. 4a–c) refer to a “snapshot” of a single configuration, while Fig. 4e,f shows averages over the 40 realizations.

## Supplementary Information

Supplementary Information.

## Data Availability

The raw data generated and analyzed as a part of this study are available from the corresponding author upon reasonable request.
