# Supplementary material for: Field-induced vortex-like textures as a probe of the critical line in reentrant spin glasses
Source: Sci Rep. 2021 Oct 21;11:20753. doi: 10.1038/s41598-021-99860-2 (PMC8531314; doi:10.1038/s41598-021-99860-2)
Supplement: Supplementary file 1 — Supplementary Information. [file 41598_2021_99860_MOESM1_ESM.pdf]

Supplementary Information for

"Field-induced vortex-like textures as a probe of the critical line in reentrant spin glasses"

N. Martin[1,*], L.J. Bannenberg[2], M. Deutsch[3], C. Pappas[2], G. Chaboussant[1], R. Cubitt[4], and I. Mirebeau[1]

[1]Université Paris-Saclay, CEA, CNRS, Laboratoire Léon Brillouin, CEA Saclay 91191 Gif-sur-Yvette, France
[2]Faculty of Applied Science, Delft University of Technology, 2629 JB Delft, the Netherlands
[3]Université de Lorraine, CNRS, CRM2, Nancy, France
[4]Institut Laue Langevin, BP156, F-38042 Grenoble, France
[*]nicolas.martin@cea.fr

ABSTRACT

In this supplement, we provide information concerning the synthesis and structural characterization of the $(Fe_{1-x}Mn_x)_{75}P_{16}B_6Al_3$ samples used in this study (Sec. 1). Composition-dependence of the magnetization and AC susceptibility data is presented in Sec. 2. In Sec. 3, we describe the strategy used to scale and analyze the small-angle neutron scattering (SANS) data. Finally, the details of our Monte Carlo simulations on systems with varying antiferromagnetic bond concentration are given in Sec. 4.

1 Samples synthesis and structural characterization

Amorphous samples of $(Fe_{1-x}Mn_x)_{75}P_{16}B_6A_3$ ($0.22 \leq x \leq 0.41$), herafter named "a-$Fe_{1-x}Mn_x$", were prepared using the "wheelbarrow" technique, which consists in casting molten alloy with the desired composition on a spinning wheel, by J. Bigot (Centre d'Études de Chimie—Metallurgie, Vitry sur Seine). Being a strong neutron absorber, ^{10}B ($\sigma_{\rm abs} = 3835$ barn) was replaced with isotopic ^{11}B ($\sigma_{\rm abs} = 0.0055$ barn). Samples were cut in foils of about 1 cm^2 surface with thicknesses varying from 30-70 μm. These foils were then piled up in order to increase the total sample thickness and yield a large enough sample mass for the small-angle neutron scattering (SANS) experiments (see Sec. 3).

Density – The density $d_{\rm a-Fe_{1-x}Mn_x}$ of these materials is an important value, allowing to calibrate the magnetization and SANS data. However, it is rather difficult to measure it directly, given the small thickness of the foils. It can nevertheless be estimated using the *random close packing* approximation in a hard-sphere model. The maximum density is 64 % of that of the densest crystalline arrangement, namely fcc, with a compacity of 0.74. Taking the atomic masses $m_{\rm n}$ (with $n = \{$Fe, Mn, P, B, Al$\}$) into account, one gets:

$$d_{\rm a-Fe_{1-x}Mn_x} = \frac{0.75 \cdot [(1-x)\, m_{\rm Fe} + x m_{\rm Mn}] + 0.16 m_{\rm P} + 0.06 m_{\rm B} + 0.03 m_{\rm Al}}{m_{\rm Fe}} \cdot \frac{0.64}{0.74} \cdot d_{\rm fcc\,Fe} \quad , \tag{1}$$

where $d_{\rm fcc\,Fe}$ = 8.879 g·cm^{-3} is the density of fcc Fe. Whenever relevant, Eq. 1 is used to scale the data presented in the main text and this supplementary material.

Amorphous nature of the samples – The amorphous nature of the samples can be assessed using neutron diffraction. As shown in Fig. 1a in the case of a-$Fe_{0.765}Mn_{0.235}$, the *normalized structure factor* $S(Q)/S(Q_{\rm max})$ (where $Q_{\rm max}$ is the largest momentum transfer reached in the experiment) lacks Bragg reflections and is characteristic of an amorphous (liquid-like) compound. The small-Q region of the pattern (see Fig. 1b) is marked by an upturn, which is well-described by a power law of the form $a_{\rm p}/Q^p$ with an exponent $p = 2.97(12)$ (typical of surface roughness, yielding $p = 3$). The presence of a prepeak, before the main structural one, indicates a possible clustering of the main chemical specie. Its intensity is however magnified due to the fact that the scattering lengths of the main constituents (i.e., Fe and Mn) have opposite signs.

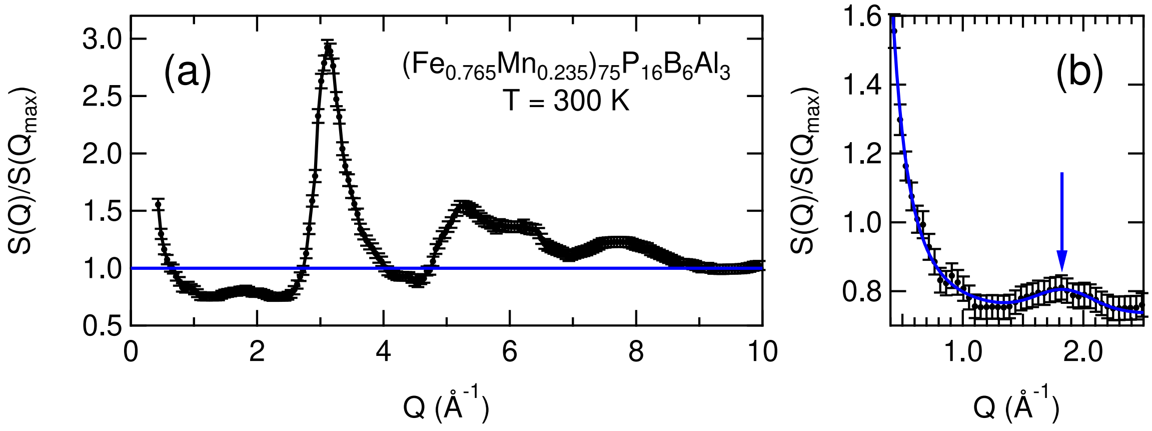

Figure 1. **(a)** Diffraction pattern of a-$Fe_{0.765}Mn_{0.235}$ (taken from Mirebeau[1]). Data is normalized such that $S(Q) \to 1$ in the limit of large Qs. **(b)** Zoom into the low Q part of the diffraction pattern, showing the prepeak at $Q \approx 1.7$ Å^{-1}. Line is a fit of a power law to the data (see text).

The *pair distribution function* (PDF) $g(r)$ is obtained from $S(Q)/S(Q_{\max})$ using

$$g(r) = 1 + \frac{1}{2\pi^2 \rho_0 r} \int_0^{Q_{\max}} Q \left[\frac{S(Q)}{S(Q_{\max})} - 1 \right] \sin(Qr)\, dQ \quad , \tag{2}$$

where ρ_0 is the atomic number density. Finally, one obtains the *radial distribution function* (RDF) $\rho(r)$ from the PDF via

$$\rho(r) = 4\pi \rho_0 r^2 g(r) \tag{3}$$

This procedure allows determining the coordination numbers z_n, *i.e.* the number of atoms in the nth shell surrounding any central atom, by integrating $\rho(r)$ within the r-range bounded by its first two minima (shaded region in Fig. 2b). For $n = 1$, we find $z_1 \approx 10.6$ and $r_1 \approx 2.6$ Å, in good agreement with results obtained on amorphous Fe powder[2] and liquid Fe[3]

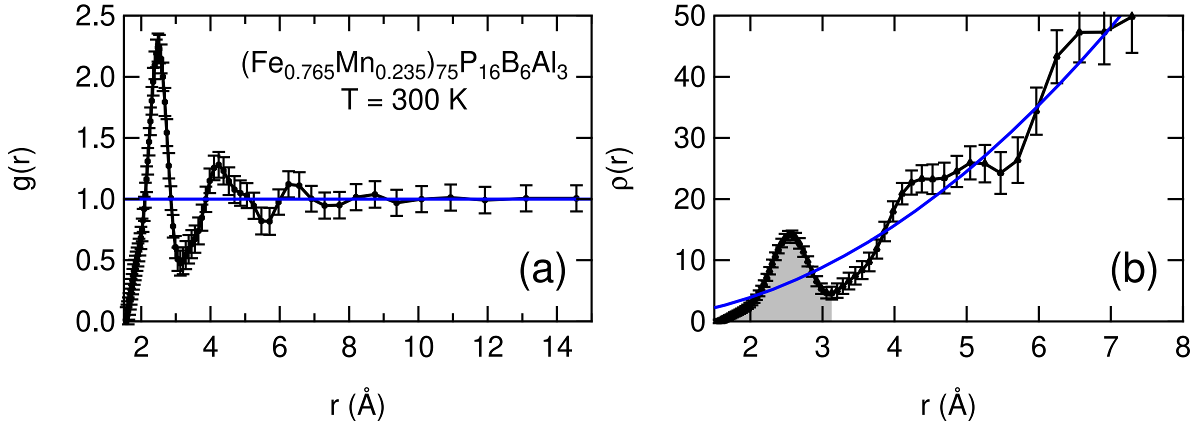

Figure 2. **(a)** Pair distribution function $g(r)$ and **(b)** radial distribution function $\rho(r)$ calculated from the data of Fig. 1.

2 Macroscopic magnetic properties

2.1 Magnetization

The field-dependences of the magnetization M of the a-$Fe_{1-x}Mn_x$ samples were measured using a MPMS-XL 5T Quantum Design SQUID magnetometer. The samples were zero-field cooled from $T \gg T_C, T_F$ down to 5 K, and specific care was taken to avoid the presence of a residual field. Subsequently, the measurements were performed by stepwise increasing the magnetic field. Samples masses of the order of several mg were used in order to be able to accurately scale the magnetic moment in Bohr magneton per formula unit (μ_B/f.u.). As shown in Fig. 3a, the field value at which magnetization reaches quasi-saturation (H_0) increases with increasing x, underscoring the increasing magnetic frustation. In all cases, M retains a finite slope up to the largest fields, as a result of the gradual collapse of the vortex-like textures located around the AFM pairs (see main text). In this regime, M is well-described by a law of the form $M \approx (\mu_0 H_{\mathrm{int}})^{1/3}$ (Fig. 3b). This property is used in the main text to discuss the possible scaling law governing the field-evolution of the observed nanoscopic magnetic textures. We can also define the saturation field H_0, at which M acquires the $(\mu_0 H_{\mathrm{int}})^{1/3}$-dependence, and compare it with the H_0 extracted from the SANS scaling laws, *i.e.* the field above which maxima in the transverse scattering cross section σ_T can be defined (Fig. 3a of main text). The good correlation between these values is illustrated by Fig. 3c.

The Arrott plots computed from the data of Fig. 3a are shown in Fig. 3d. All studied samples with $x < x_C$ display a non-zero spontaneous magnetization M_0. Theses values are plotted in Fig. 3e. The extrapolated value of M_0 for $x \to 0$ compares well with literature values for crystalline Fe (Shull[4]), and amorphous $Fe_{75}B_{25}$ (Cowlam & Carr[5]), Fe (Grinstaff *et al.*[6]) and $Fe_{75}P_{12.5}B_{12.5}$ (Durand & Yung[7]), see Fig. 3.

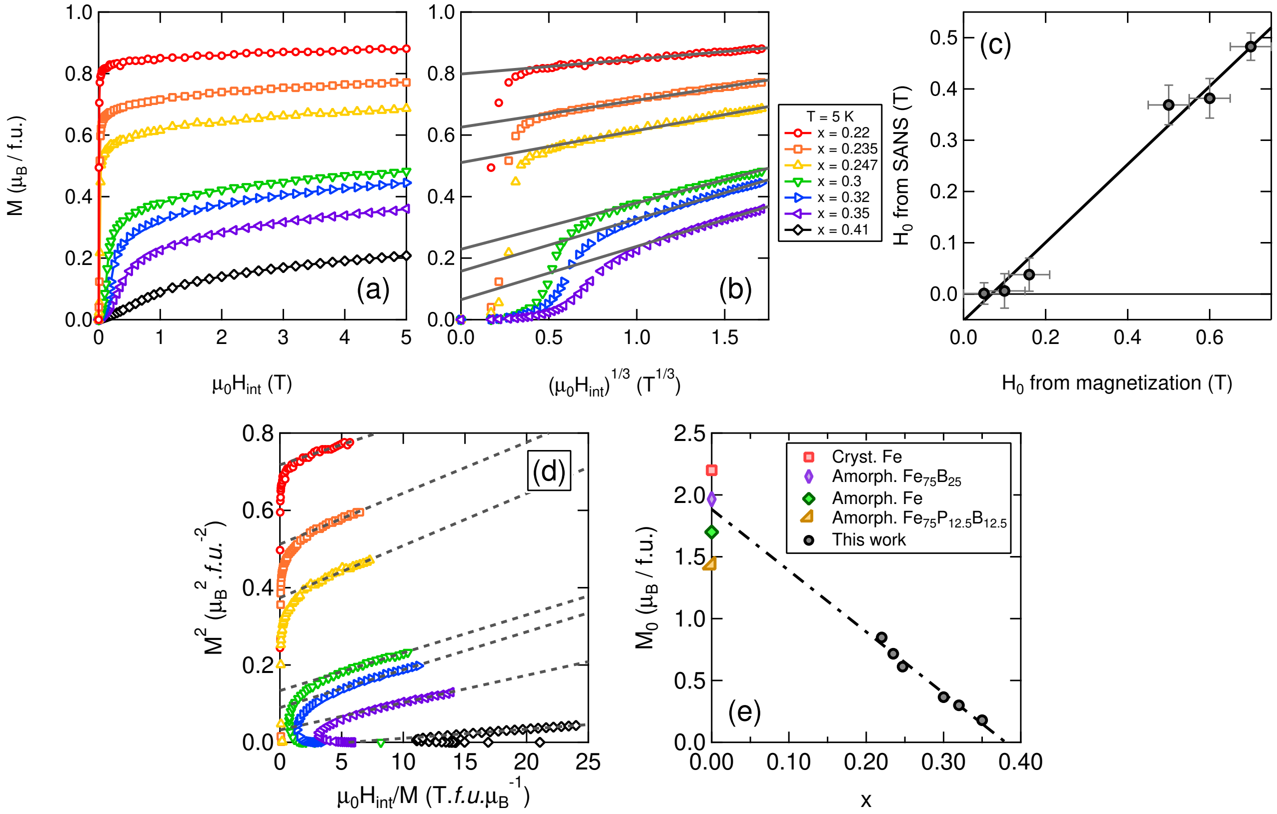

Figure 3. Macroscopic magnetization M of the studied a-FeMn samples as a function of **(a)** $\mu_0 H_{\mathrm{int}}$ and **(b)** $(\mu_0 H_{\mathrm{int}})^{1/2}$. **(c)** Saturation field value H_0 deduced from SANS data as a function of H_0 deduced from magnetization curves. **(d)** Low temperature Arrott plots (T = 5 K). **(e)** x-dependence of the saturated moment, inferred from the Arrott plots. It vanishes for $x \approx 0.38$ and extrapolates to a value of $\approx 1.86\,\mu_B \cdot \mathrm{f.u.}^1$ for $x \to 0$. This value is compared with previous results for crystalline and amorphous Fe.

2.2 ac-susceptibility

The magnetic phase diagram presented in the main text was infered through AC susceptibility measurements, performed using a Quantum Design Dynacool 9 T Physical Properties Measurement Systems (PPMS) at the Laboratoire Léon Brillouin. Unless otherwise stated, data presented in this section were measured under an AC field of 1 kHz frequency and 10 Oe amplitude, in zero-applied static field. In order to suppress demagnetizing field effects, we have cut the individual foils into rectangular pieces, having a height to width ratio close to 2 in each case. This however lead to very small samples masses (< 100 μg) and therefore to relatively weak signals. The AC field was applied in the sample plane, along its larger dimension. In what follows, we show how phase boundaries are deduced from maxima in $d\chi'(T)/dT$ curves.

Table 1. Dimensions of the a-$Fe_{1-x}Mn_x$ samples used for the AC susceptibility measurements.

x	Height (mm)	Width (mm)
0.22	4.7	2.3
0.235	4.4	2.4
0.247	4.2	2.2
0.3	4.6	2.2
0.32	5.0	2.2
0.35	4.3	1.8
0.41	4.0	2.1

The temperature-dependence of the real (χ') and imaginary (χ'') part of the AC susceptibility of a-$Fe_{1-x}Mn_x$ samples with $0.22 \leq x \leq 0.41$ is shown in Figs. 4-6. For compositions $x \leq 0.32$, the Curie (T_C) and spin freezing (T_F) temperatures are easily evidenced by well-separated extrema in the first temperature derivative of χ' (Figs. 4 and 5), (*i.e.* using the same procedure as used by Yeshurun[8]). The $x = 0.35$ case, located very close to the RSG-SG thershold composition $x_C \approx 0.36$, is more difficult to anlyze. At first glance, its ac-susceptibility is very close to that of the pure SG with $x = 0.41$ (Fig. 6). However, a modest field has a large impact on the $d\chi'/dT$ of the $x = 0.35$ sample as opposed to the $x = 0.41$ one. This suggests a remanence of ferromagnetism in the former, disappearing in the latter case.

In all cases, χ'' peaks at temperatures slightly higher than T_F, while it falls off to ≈ 0 around T_C. Since T_F is known to depend on the ac-field frequency f_{ac}, we have measured the temperature-dependence of the ac-susceptibility of all samples for 100 Hz $\leq f_{ac} \leq$ 10 kHz (not shown). The various T_F shown in Fig. 1a of the main text are the results of extrapolations to $f_{ac} = 1$ Hz.

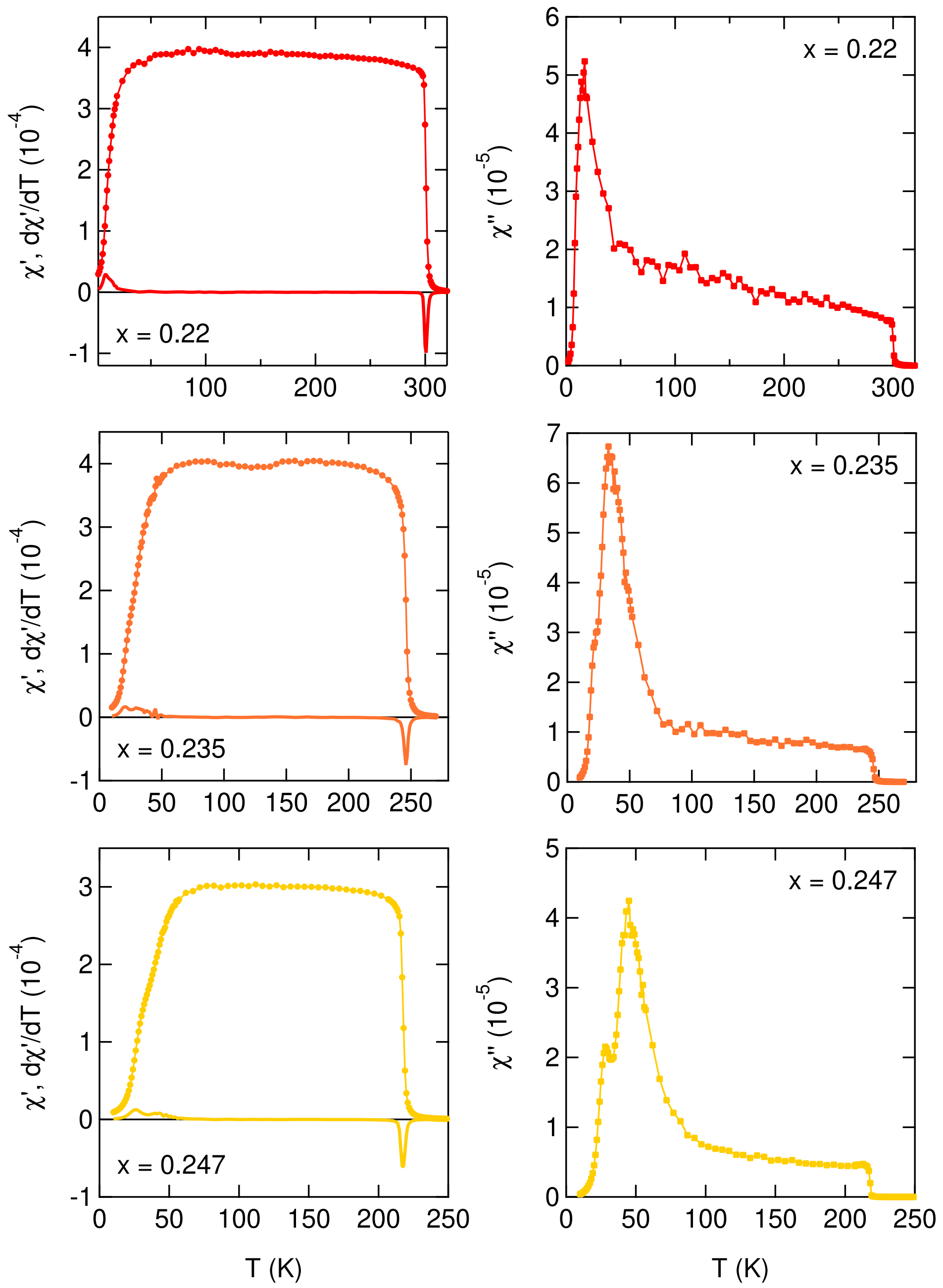

Figure 4. Zero-field AC suceptibility of a-$Fe_{1-x}Mn_x$ samples with $0.22 \leq x \leq 0.247$ in zero applied field – (Left column) Real part of the AC susceptibility χ' (dots) and its first temperature derivative $d\chi'/dT$ (solid lines). **(Right column)** Imaginary part of the AC susceptibility χ''. Data is normalized to samples' surfaces, such that χ' and χ'' are expressed in emu.g^{-1}.Oe^{-1}.mm^{-2}, and $d\chi'/dT$ in emu.g^{-1}.Oe^{-1}.mm^{-2}.K^{-1}.

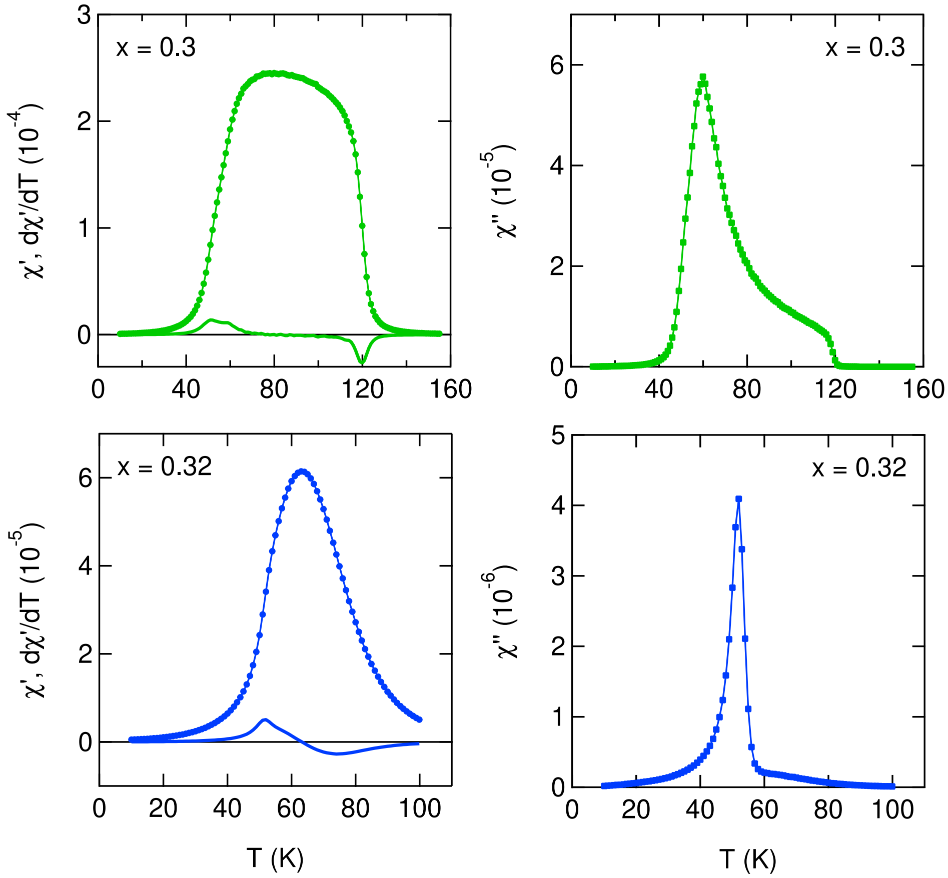

Figure 5. AC suceptibility of a-$Fe_{1-x}Mn_x$ samples with x = 0.3 and 0.32 in zero applied field – (Left column) Real part of the AC susceptibility χ' (dots) and its first temperature derivative $d\chi'/dT$ (solid lines). **(Right column)** Imaginary part of the AC susceptibility χ''. Data is normalized to samples' surfaces, such that χ' and χ'' are expressed in emu.g^{-1}.Oe^{-1}.mm^{-2}, and $d\chi'/dT$ in emu.g^{-1}.Oe^{-1}.mm^{-2}.K^{-1}.

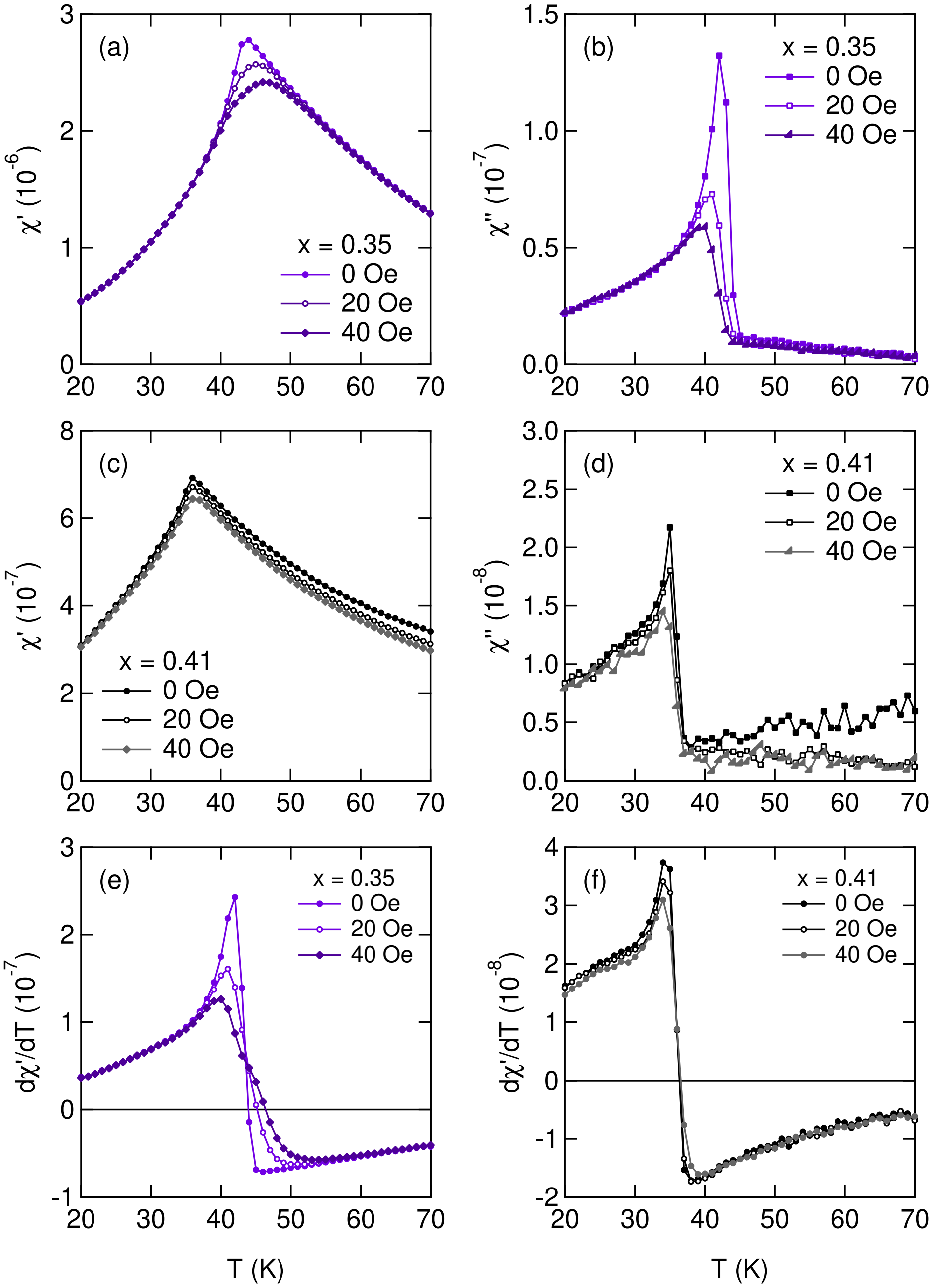

Figure 6. AC suceptibility of a-$Fe_{1-x}Mn_x$ samples with x = 0.35 and 0.41 in zero and small applied field – (a,b) Real (χ') and imaginary (χ'') part of the AC susceptibility of a-$Fe_{0.65}Mn_{0.35}$. **(c,d)** Real (χ') and imaginary (χ'') part of the AC susceptibility of a-$Fe_{0.59}Mn_{0.41}$. **(e,f)** First temperature-derivative of χ'. While a weak applied field has a substantial effect of the high-temperature minimum in $d\chi'/dT$ for the x = 0.35 sample, it remains unchanged in the x = 0.41 case. In all panels, data is normalized to samples' surfaces, such that χ' and χ'' are expressed in emu.g^{-1}.Oe^{-1}.mm^{-2}, and $d\chi'/dT$ in emu.g^{-1}.Oe^{-1}.mm^{-2}.K^{-1}.

3 Small-angle neutron scattering

3.1 Experimental geometry

The small-angle neutron scattering (SANS) experiment described in the main text was performed on the PAXY instrument at the Orphée reactor (LLB, Gif-sur-Yvette, France). We have used a standard pinhole geometry, with parameters given in Tab. 2.

Table 2. Parameters used for the SANS experiment on the PAXY instrument.

Neutron wavelength	$\rightarrow$	4 and 6 Å
Source aperture (diameter)	$\rightarrow$	16 mm
Collimation length	$\rightarrow$	2.25 m
Sample aperture (diameter)	$\rightarrow$	5 mm
Sample-to-detector distance	$\rightarrow$	2.8 m

A horizontal magnetic field $H = 0-4\,\mathrm{T}$ was supplied using an Oxford 10 T cryomagnet (Spectromag SM4000). Throughout the experiment, field was applied perpendicular to the beam direction in order to optimally resolve the azimuthal asymmetry of the magnetic scattering (see Eq. 1 of main text). Samples were wrapped into a thin Al foil, sandwiched between two Cd slabs (to suppress background and get a well-defined sample surface with 5 mm diameter) and stuck on a Cu frame (to insure a good thermal conduction with the thermometer).

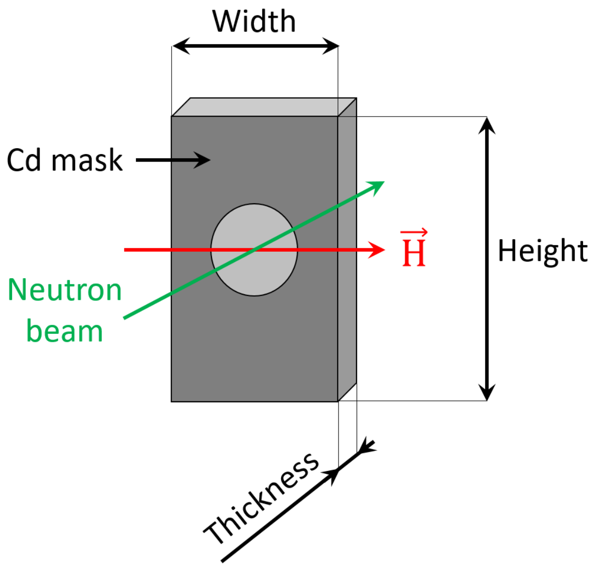

Figure 7. Experimental sample geometry used during the SANS experiment. The horizontal magnetic field $\vec{H}$ was applied transverse to the largest sample dimension.

For practical reasons, the magnetic field could not be applied along the largest dimensions of the samples (see Fig. 7). In order to obtain scaling laws depending on the *internal* magnetic field $\mu_0 H_{\mathrm{int}}$ experienced by the samples, we have calculated the demagnetizing field using magnetization data presented in Sec. 2 (for which demagnetizing field was negligible) and the demagnetization factor for a very flat ellipsoid given by Osborn[9] (Eq. 2.24). The latter approximation is justified in view of the effective sample dimensions (Tab. 3).

Table 3. Dimensions of the a-FeMn samples used in the SANS study.

x	Thickness (mm)	Width (mm)	Height (mm)
0.22	3.0	9.1	30.9
0.235	2.1	8.0	36.6
0.247	2.3	8.9	38.0
0.3	1.6	8.6	37.7
0.32	1.5	9.0	21.6
0.35	1.9	9.2	33.8
0.41	4.9	8.7	33.0

3.2 Data correction

In order to get scattering cross sections in absolute units, we follow the usual data reduction procedure. First, the contribution from the environment and direct beam are removed using

$$I^{\mathrm{sub}}(Q) = \frac{I(Q)/t(Q) - \frac{t(Q)}{t_{\mathrm{empty\ cell}}(0)} \cdot I_{\mathrm{empty\ cell}}(Q)/t_{\mathrm{empty\ cell}}(Q)}{\Omega(Q)} \quad , \tag{4}$$

where $I(Q)$, $t(Q)$ and $\Omega(Q)$ are the Q-dependent raw intensities, sample transmissions and solid angles subtended by the corresponding detector pixels[10]. In Eq. 4, the subscript "empty cell" denotes a measurement performed using the same sample holder assembly as for the sample (including the Al foil).

This subtraction procedure is applied to the SANS from the samples and from a Ni single crystal. The latter is used to transform the observed intensities in absolute cross sections, according to

$$\sigma(Q) = \frac{I^{\mathrm{sub}}_{\mathrm{a-FeMn}}(Q) \cdot t_{\mathrm{Ni}}(0) \cdot d_{\mathrm{Ni}} \cdot e_{\mathrm{Ni}}}{\langle I^{\mathrm{sub}}_{\mathrm{Ni}}(Q)\rangle \cdot t_{\mathrm{a-FeMn}}(0) \cdot d_{\mathrm{a-FeMn}} \cdot e_{\mathrm{a-FeMn}}} \cdot \sigma^{\mathrm{inc}}_{\mathrm{Ni}} \quad , \tag{5}$$

where t, d and e denote transmission, atomic density (see Sec. 1) and sample thickness (see Tab. 3), respectively, while $\sigma^{\mathrm{inc}}_{\mathrm{Ni}} = 5.2/4\pi$ barn.sr^{-1} is the incoherent scattering cross section of Ni ($\langle ... \rangle$ denotes the average over the detector surface, where incoherent scattering of Ni is expected to be flat).

3.3 Effect of a cooling field on the SANS of the x = 0.22 sample

As noted in seminal experimental[11] and theoretical[12] studies of spin glasses, the anisotropy field maintaining the remanent magnetization in the direction of an initial applied field strongly depends of the elements composing the studied material. This comes from additional terms in the Ruderman-Kittel-Kasuya-Yosida (RKKY) interaction for atoms with large spin-orbit coupling (such as Au, Pt, *etc.*), leading to Dzyaloshinskii-Moriya (DM) anisotropy of unidirectional type. In SG and RSG, this DM anisotropy modifies the torque magnetization, the shape and position of the hysteresis cycle, and the effect of a cooling field.

When the DM term is small, an applied field causes the magnetization to rotate "rigidly" against the DM-induced domain anisotropy. On the other hand, when the DM term is strong, domain configurations may relax, yielding a distribution in the strength of the resulting frictional torque. In the former case, this leads to displaced narrow hysteresis loops under field cooling (FC) conditions while, in the latter case, one observe undisplaced broadened loops, independent of the cooling conditions.

Rotational magnetization measurements in a-FeMn ($x = 0.235$) by Goeckner & Kouvel[13] have revealed that this system is much less "rigid" than *e.g.* $Ni_{1-x}Mn_x$, where the anisotropy field is much smaller[14].

To test the influence of the DM anisotropy on the vortex size, we have checked the effect of a cooling field on the scaling law of their average size *vs* field $Q_{\max} = f(\mu_0 H_{\mathrm{int}})$ (see Eq. 3 of main text) in both systems[15]. The result is shown on Fig. 8, clearly demonstrating that $Q_{\max}$ is progressively shifted upwards by a cooling field in $Ni_{0.81}Mn_{0.19}$ while it remains unchanged in the case of a-$Fe_{0.78}Mn_{0.22}$. In other words, FC induces an extra magnetic field which reduces the vortex size in NiMn, whereas it has no effect in a-FeMn. This shows that the DM anisotropy indeed plays a role on the vortex landscape, as it does on the magnetization, and underscores the strong relation between the spin vortices and the underlying ferromagnetic vacuum.

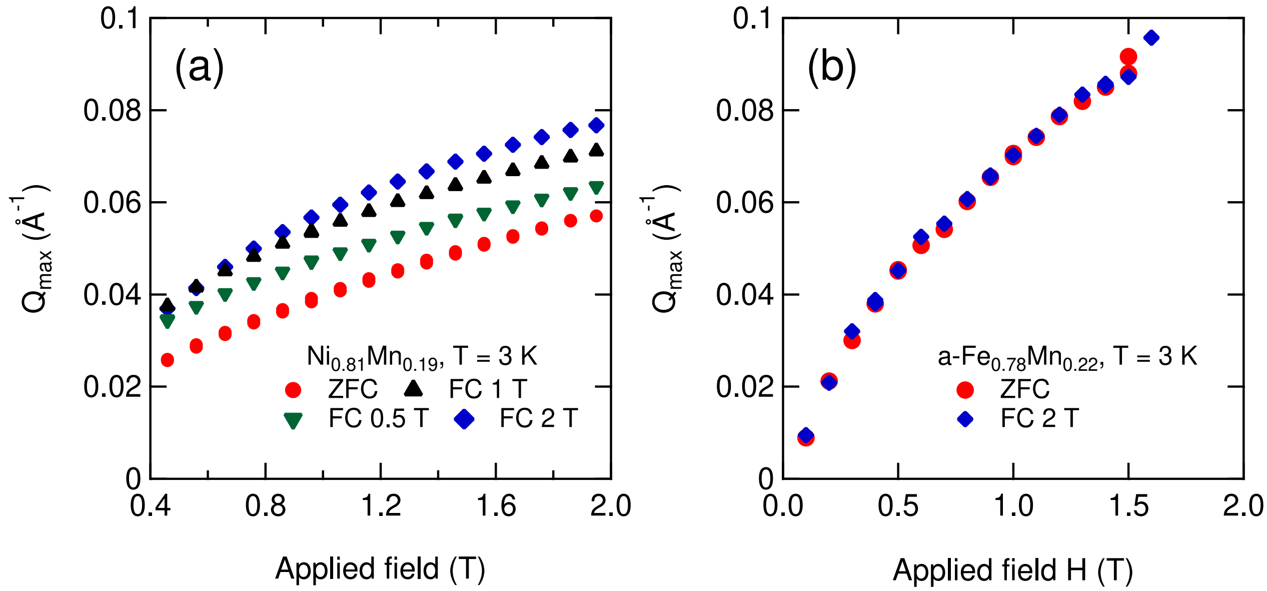

Figure 8. Field-dependence of the position $Q_{\max}$ of the maximum in transverse magnetic cross section $\sigma_T(Q)$ for **(a)** $Ni_{0.81}Mn_{0.19}$ single crystal[16] and **(b)** a-$Fe_{0.78}Mn_{0.22}$.

4 Monte Carlo simulations

4.1 Model and parameters of the simulations

Monte Carlo simulations presented in the main text have been performed using a standard local update algorithm on a square lattice containing $L \times L = 10^4$ spins, allowed to point in all directions of the 3d space (*i.e.* we work out a 2d Heisenberg model). "Impurity" spins are randomly spread over the matrix with the desired concentration x to form a binary alloy with composition $A_{1-x}B_x$. These impurities emulate the Mn ions and are antiferromagnetically (AFM) coupled with other first neighbor impurity spins ($J = -1$), while all other couplings are ferromagnetic (FM, $J = +1$).

In order to obtain reasonable convergence times, we have used the "adaptative" algorithm proposed by Alzate-Cardona *et al.*[17]. Trial moves of individual spin orientations are performed within a cone having an opening angle $\nu = 60°$ with respect to the initial spin orientation. After each Monte Carlo step (MCS, corresponding to 10^4 moves), ν is modified according to $\nu_{\mathrm{new}} \to \nu_{\mathrm{old}} \times f$ with

$$f = \frac{0.5}{1 - R_{\mathrm{old}}} \quad , \tag{6}$$

where R_{old} is the acceptance rate observed during the previous MCS. This procedure allows keeping the average acceptance rate of the algorithm close to 50 %, thereby leading to a relatively quick convergence. For each move, the classical energy

$$\mathscr{H} = -\sum_{ij} J_{ij}\,\mathbf{S}_i \cdot \mathbf{S}_j - 0.672\,H \sum_i S_i^z \tag{7}$$

is calculated, where $\mathbf{S}_{i,j}$ are classical Heisenberg spins with $|\mathbf{S}_{i,j}| = 1$, J_{ij} are nearest neighbor (NN) exchange constants with $|J_{ij}| = 1$ and the magnetic field H is applied along the z direction. The first sum in Eq. 7 runs over NN pairs. The factor 0.672 $(= \mu_B/k_B)$ appearing in Eq. 7 allows getting energies in K for magnetic fields expressed in T. The acceptance of each trial move is tested against the Maxwell-Boltzmann statistics $\exp(-\Delta E/T)$, where ΔE is the energy difference between the "old" and "new" configurations). For each studied composition, we performed between 24 and 40 realizations of the following sequence:

1. Random generation of the spin matrix at $T/J = 2$ and $H = 0$ with a chosen "impurity" concentration x,
2. Slow cooling down to $T/J = 0.01$ at $H = 0$ (500 MCS per step), in steps of $T/J = 0.1$,
3. Field sweep in the $H/J = 0-1$ range, in small steps of $\Delta H/J = 0.01$ (500 MCS per step).

4.2 Data analysis & x-dependences

In order to compare the result of our MC simulations with that of the SANS experiment, we compute the square of the Fourier transform of the spin matrices which is formally equivalent to the scattering cross section in the absence of correlation between defects (see Eq. 2 of main text). As in the experimental case, one can separate correlation functions for the transverse (T) and longitudinal (L) magnetization. Illustrative examples are shown in Fig. 4 of main text. Note that the explored momentum range is bounded downwards by the size of the spin maps (100×100) and upwards by the nearest neighbor (NN) distance, but remains much larger than the one covered by SANS (see below).

In addition to the $x = 0.23$ ($c_{\mathrm{AFM}} \approx 0.05$)-case discussed in the main text, we have also explored different concentrations in order to check the applicability of our primitive simulations when x (and, hence, $c_{\mathrm{AFM}} = x^2$) changes within the weakly frustrated side of the phase diagram (*i.e.*, for $\approx 0.05 \lesssim c_{\mathrm{AFM}} \lesssim 0.26$). The obtained magnetization curves are plotted in Fig. 9a, showing the same behavior as a function of increasing frustration as the experimental ones (Fig. 1b of main text and Fig. 3a of this supplement). We have also determined the scaling laws of Q_{max} as a function of the applied magnetic field (Fig. 9b). A global fit of Eq. 3 of main text to the data yields an exponent $\gamma = 0.49(1)$ that is slightly different from the experimental value (≈ 0.39). Of course, our simulations are only taking NN interactions on a square lattice into account. It is not surprising that the scaling laws are renormalized by the effect of an increased number of first neighbors (see Sec. 1) and longer-ranged interactions in the real amorphous metallic samples. However, the simulated scaling laws are very similar to the experimental ones in the sense that the scaling paramter κ, monitoring the "stiffness" of these curves, increases linearly with x in agreement with the experiment (Fig. 9c). Taking these results together, we find that such a simplified model already captures many experimental features. Moreover, the MC simulations show that the scaling laws are indeed verified up to large concentrations of AF interactions, and to high values of the H/J ratio.

This simple model could of course be extended to account *e.g.* for the behavior at the RSG-SG threshold, taking more realistic values for the moments, exchange constants and atomic connectivities.

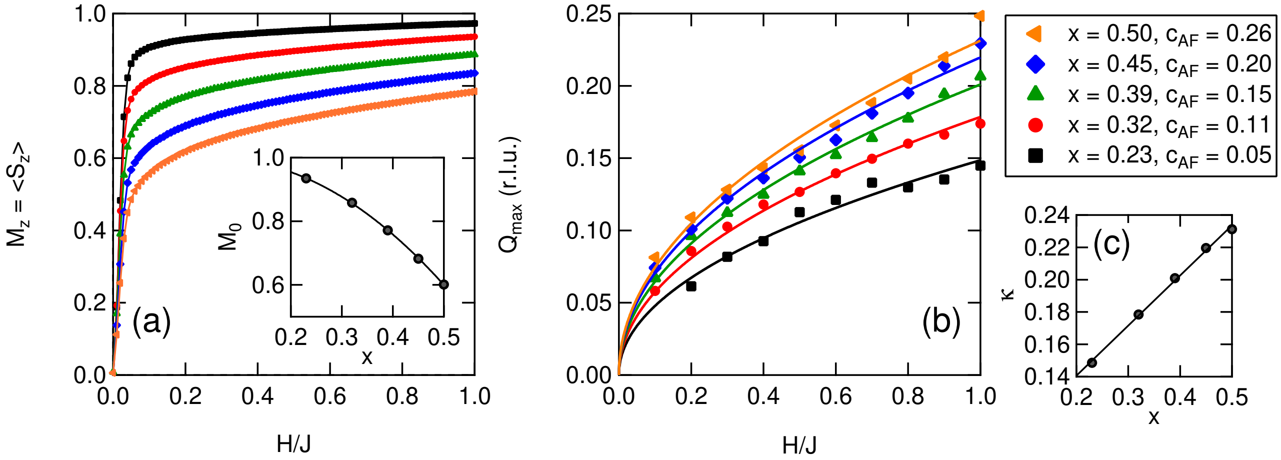

Figure 9. **(a)** x-dependence of the magnetization curves derived from the MC simulations. Inset shows the x-dependence of the spontaneous magnetization M_0, determined from Arrot plots of the field-dependent magnetization $M_z(H)$. **(b)** Field-dependence of the position Q_{max} of the maximum in $|F_T(Q)|^2$ for each studied compositions. Solid lines are results of a global fit of Eq. 3 of main text to the data. **(c)** x-dependence of the scaling parameter κ, extracted from a fit of Eq. 3 of main text to the data of panel **(b)**.

As discussed above, the MC simulations allow extending the explored Q-range to values $Q \approx 1$, sensitive to the smallest interatomic distance. This is an interesting asset, since one can expect in this Q-range (which naturally evades the SANS window), a growing AFM contribution to the scattering pattern as x increases. As shown in Fig. 10, where we have selected field values such that Q_{max} stays constant for different x, this is indeed the case.

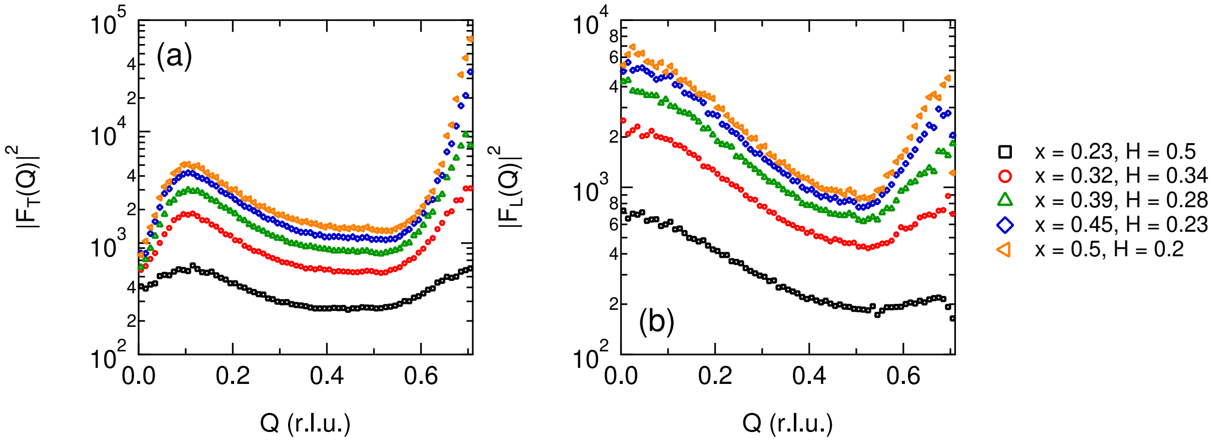

Figure 10. Squared Fourier transforms of the transverse **(a)** and longitudinal **(b)** spin correlations for different x and magnetic field values, chosen to yield a constant Q_{max} (see text).

References

1. Mirebeau, I. *Contribution à l'Etude des Systèmes Ferromagnétiques Réentrants à Tendance Verre de Spin*. Ph.D. thesis, Université de Paris-Sud (1987).
2. Bellissent, R., Galli, G., Grinstaff, M. W., Migliardo, P. & Suslick, K. S. Neutron diffraction on amorphous iron powder. *Phys. Rev. B* **48**, 15797–15800, DOI: 10.1103/PhysRevB.48.15797 (1993).
3. Waseda, Y. & Suzuki, K. Atomic distribution and magnetic moment in liquid iron by neutron diffraction. *physica status solidi (b)* **39**, 669–678, DOI: https://doi.org/10.1002/pssb.19700390235 (1970). https://onlinelibrary.wiley.com/doi/pdf/10.1002/pssb.19700390235.
4. Shull, C. G. & Wilkinson, M. K. Neutron diffraction studies of the magnetic structure of alloys of transition elements. *Phys. Rev.* **97**, 304–310, DOI: 10.1103/PhysRev.97.304 (1955).
5. Cowlam, N. & Carr, G. E. Magnetic and structural properties of Fe-B binary metallic glasses. I. Variation of magnetic moment with composition. *J. Phys. F: Met. Phys.* **15**, 1109–1116, DOI: 10.1088/0305-4608/15/5/016 (1985).
6. Grinstaff, M. W., Salamon, M. B. & Suslick, K. S. Magnetic properties of amorphous iron. *Phys. Rev. B* **48**, 269–273, DOI: 10.1103/PhysRevB.48.269 (1993).
7. Durand, J. & Yung, M. *Electronic and Magnetic Properties of Amorphous Fe-P-B Alloys*, 275–288 (Springer US, Boston, MA, 1977).
8. Yeshurun, Y., Salamon, M. B., Rao, K. V. & Chen, H. S. Critical phenomena in amorphous ferromagnetic and spin-glass alloys. *Phys. Rev. B* **24**, 1536–1549, DOI: 10.1103/PhysRevB.24.1536 (1981).
9. Osborn, J. A. Demagnetizing factors of the general ellipsoid. *Phys. Rev.* **67**, 351–357, DOI: 10.1103/PhysRev.67.351 (1945).
10. Brûlet, A., Lairez, D., Lapp, A. & Cotton, J.-P. Improvement of data treatment in small-angle neutron scattering. *J. Appl. Crystallogr.* **40**, 165–177, DOI: 10.1107/S0021889806051442 (2007).
11. Préjean, J., Joliclerc, M. & Monod, P. Hysteresis in cumn : The effect of spin orbit scattering on the anisotropy in the spin glass state. *J. Phys. France* **41**, 427–435, DOI: 10.1051/jphys:01980004105042700 (1980).
12. Fert, A. & Levy, P. M. Role of anisotropic exchange interactions in determining the properties of spin-glasses. *Phys. Rev. Lett.* **44**, 1538–1541, DOI: 10.1103/PhysRevLett.44.1538 (1980).
13. Goeckner, H. & Kouvel, J. Rotational spin-glass properties of amorphous $(Fe_{1-x}Mn_x)_{75}P_{16}B_6Al_3$. *J. Appl. Phys.* **70**, 6089–6091, DOI: 10.1063/1.350055 (1991). https://doi.org/10.1063/1.350055.
14. Kouvel, J. S., Abdul-Razzaq, W. & Ziq, K. Ferro-spin-glass domain model for disordered Ni-Mn. *Phys. Rev. B* **35**, 1768–1775, DOI: 10.1103/PhysRevB.35.1768 (1987).
15. Mirebeau, I. *et al.* Frustrated skyrmions in reentrant spin glasses: checking the Skyrmion lattice by SANS (2015). DOI: 10.5291/ILL-DATA.5-42-409.
16. Mirebeau, I. *et al.* Spin textures induced by quenched disorder in a reentrant spin glass: Vortices versus "frustrated" skyrmions. *Phys. Rev. B* **98**, 014420, DOI: 10.1103/PhysRevB.98.014420 (2018).
17. Alzate-Cardona, J. D., Sabogal-Suárez, D., Evans, R. F. L. & Restrepo-Parra, E. Optimal phase space sampling for Monte Carlo simulations of Heisenberg spin systems. *J. Physics: Condens. Matter* **31**, 095802, DOI: 10.1088/1361-648x/aaf852 (2019).
